# Effective connectivity of working memory performance: a DCM study of MEG data

**DOI:** 10.3389/fnhum.2024.1339728

**Published:** 2024-03-04

**Authors:** Aniol Santo-Angles, Ainsley Temudo, Vahan Babushkin, Kartik K. Sreenivasan

**Affiliations:** ^1^Division of Science and Mathematics, New York University Abu Dhabi, Abu Dhabi, United Arab Emirates; ^2^Center for Brain and Health, New York University Abu Dhabi, Abu Dhabi, United Arab Emirates

**Keywords:** dynamic causal model (DCM), working memory, magnetoencephalography (MEG), effective connectivity, binding problem

## Abstract

Visual working memory (WM) engages several nodes of a large-scale network that includes frontal, parietal, and visual regions; however, little is understood about how these regions interact to support WM behavior. In particular, it is unclear whether network dynamics during WM maintenance primarily represent feedforward or feedback connections. This question has important implications for current debates about the relative roles of frontoparietal and visual regions in WM maintenance. In the current study, we investigated the network activity supporting WM using MEG data acquired while healthy subjects performed a multi-item delayed estimation WM task. We used computational modeling of behavior to discriminate correct responses (high accuracy trials) from two different types of incorrect responses (low accuracy and swap trials), and dynamic causal modeling of MEG data to measure effective connectivity. We observed behaviorally dependent changes in effective connectivity in a brain network comprising frontoparietal and early visual areas. In comparison with high accuracy trials, frontoparietal and frontooccipital networks showed disrupted signals depending on type of behavioral error. Low accuracy trials showed disrupted feedback signals during early portions of WM maintenance and disrupted feedforward signals during later portions of maintenance delay, while swap errors showed disrupted feedback signals during the whole delay period. These results support a distributed model of WM that emphasizes the role of visual regions in WM storage and where changes in large scale network configurations can have important consequences for memory-guided behavior.

## Introduction

Neural activity supporting working memory (WM) is distributed throughout the brain (Christophel et al., 2017; Sreenivasan and D'Esposito, 2019; Mejías and Wang, 2022), motivating the view that WM may be better understood as a distributed network function, rather than being localized to specific brain regions (Lorenc and Sreenivasan, 2021; Rezayat et al., 2022), as suggested by evidence of task-dependent reorganization of large-scale brain networks during WM (e.g., Cohen and D'Esposito, 2016). This “new” perspective of WM was anticipated by Patricia Goldman-Rakic, who pioneer the study of the neurobiological underpinnings of WM (Goldman-Rakic, 1995). Goldman-Rakic and colleagues focused on the role of prefrontal cortex in higher cognitive functions, particularly dorsolateral prefrontal cortex (dlPFC) in WM, but their work also demonstrated that dlPFC was densely connected with posterior parietal cortex, both within- and between-hemispheres (Schwartz and Goldman-Rakic, 1984; Cavada and Goldman-Rakic, 1989). Remarkably, these two regions were also connected with a widespread and distributed network of cortical and subcortical regions supporting spatially guided behavior (Selemon and Goldman-Rakic, 1988). Despite this early evidence, the majority of subsequent investigations into WM networks, particularly in human brain imaging, have focused on interactions between prefrontal and parietal cortex based on findings that (i) these regions consistently coactivate during WM tasks (Rottschy et al., 2012, 2013; Daniel et al., 2016), (ii) modulation of frontoparietal activity by TMS/tACS impacts behavioral performance (Kessels et al., 2000; Postle et al., 2006; Polanía et al., 2012; Violante et al., 2017; Tseng et al., 2018; Biel et al., 2022), and (iii) that communication between distinct frontal and parietal sites are task-dependent and content-specific (Salazar et al., 2012; Ratcliffe et al., 2022), are modulated by WM load (Crespo-Garcia et al., 2013; Syrjälä et al., 2021), and predict individual behavioral capacity in visual WM (Palva et al., 2010). Taken together, these findings strongly support the early evidence of Goldman-Rakic and colleagues that network activity involving frontal and parietal cortices may be key for understanding WM function.

There remain several unanswered questions about how network activity supports WM. First, the majority of studies about WM network function do not investigate effective connectivity between brain regions, limiting inferences about how interregional communication supports WM. Effective connectivity refers to the directed causal influence that one neural system or region exerts over another, underscoring the dynamic and experiment-dependent nature of these interactions (Friston, 2011). This concept encapsulates the notion that the strength and direction of influence between distinct brain regions can vary based on specific experimental conditions or tasks. In human imaging data, effective connectivity can be addressed using dynamic causal modeling (DCM), a Bayesian framework designed to infer, from brain activity measurements, hidden neuronal states with a neurobiological meaning, such as the context-dependent modulation of network dynamics and the differential contribution of feedforward (bottom-up) and feedback (top-down) signals (David and Friston, 2003; Stephan et al., 2010; Friston et al., 2019). Previous DCM studies using fMRI with an n-back WM task reported an association between memory load and enhanced effective connectivity in frontoparietal feedforward (Dima et al., 2014; Jung et al., 2018) and feedback signals (Heinzel et al., 2017). A DCM study of local field potentials in non-human primates during a change detection task (Pinotsis et al., 2019) also reported load-dependent modulations of effective connectivity in the frontoparietal network. Furthermore, disturbances in feedback coupling were observed when the number of items surpassed cognitive capacity, underscoring the behavioral significance of large-scale effective connectivity.

Second, network studies largely focused on dorsolateral prefrontal and posterior parietal regions while ignoring the contributions of medial prefrontal regions, despite the robust activation of superior frontal areas during the delay period (Li et al., 2022), its association with spatial WM (Courtney et al., 1998; Rottschy et al., 2012, 2013) and the pattern of structural connectivity of the superior frontal with other WM nodes in the frontoparietal networks (Briggs et al., 2020). The contributions of sensory regions have also been ignored, particularly during WM maintenance, when sensory stimulation is absent. Understanding how these regions interact with frontoparietal circuits has the potential to shed light on the debate about the role of sensory regions in WM (Xu, 2020; Lorenc and Sreenivasan, 2021), adjudicating between the sensory recruitment hypothesis (Pasternak and Greenlee, 2005; D'Esposito, 2007), which is supported by findings from human neuroimaging studies that demonstrate that visual WM contents can be decoded from visual areas (Sreenivasan et al., 2014a; Curtis and Sprague, 2021), and the countervailing view that early sensory activity during WM reflects feedback signals from parietal or frontal regions (Leavitt et al., 2017; Xu, 2020). The study of effective connectivity, particularly the contribution of feedforward/feedback signals from/to early visual areas during the delay period has the potential to make an important contribution to this debate.

Third, the behavioral relevance of WM network activity is underspecified. Behavioral relevance is often inferred from changes in network activity as a function of WM load. However, load-dependent modulations may be due to progressive deployment of neural resources in response to cognitive demands rather than WM computations *per se* (Palva et al., 2010). Even studies that do not rely on WM load differences do not discriminate between different types of behavioral errors that might emerge from distinct neural sources. Behavioral and computational evidence suggest that, when the integration or binding of visual features is required, behavioral errors consist a mixture of low accuracy errors (guess responses) and swap errors. The former arise from disrupted neural representations of individual features, while the latter appears as a disruption in the integration between features (Bays, 2016; Schneegans and Bays, 2017, 2019). Indeed, binding errors are associated with lesions in frontoparietal networks (Lugtmeijer et al., 2021), and feature binding has been associated with intraparietal sulcus activity, relatively independent from cognitive load (Gosseries et al., 2018) or stimulus identity (Cai et al., 2020). In an fMRI study with a delayed continuous report task (Mallett et al., 2022), successfully reconstructed location-specific representations from early visual cortex and intraparietal area in target trials. Interestingly, in swap trials, target location could not be reconstructed, but both areas successfully reconstructed the non-target stimulus on the location of subsequent swapped report, indicating that swap errors arise from incorrect responses being maintained in WM. While this evidence indicates that swap and low accuracy trials may have distinct origins at the meso-circuit scale, here we examine how large-scale network properties contribute to behavioral outcomes in WM.

In the current study, we examined how WM performance depends on network activity using a combination of data-driven approaches to identify key nodes in a brain-wide WM network and model-driven approaches to infer directional influences and distinguish between different types of behavioral errors. We analyzed MEG data acquired while healthy subjects performed a delayed estimation task for location, and found that this task engaged nodes in lateral and medial frontal cortex, as well as parietal and occipital cortices. We used DCM to measure effective connectivity between these nodes and found that a key element of this network was feedforward connections between visual and frontoparietal regions. Finally, discriminating between trials with high accuracy responses, low accuracy responses, and swap errors allowed us to describe how differential configurations of this network are associated with different types of behavioral errors.

## Methods

### Sample

We invited 30 healthy adults from the NYUAD community to participate in this study, recruited from a larger pool who had previously participated in a separate behavioral study with the same task. In order to ensure that we had sufficient error trials for our analyses, we only invited subjects who had symmetric swaps (see below) on at least 5% of trials in the behavioral study. The final sample included in our analyses was 26 subjects [age: 24.33 ± 4.57, range (19–37); four females, 22 males], after discarding three subjects due to poor performance (fewer than 15 correct responses in one or more conditions of interest), and another due to technical challenges transforming MEG data from sensor space to source space. All subjects were right-handed with normal or corrected-to-normal vision, and provided informed written consent in accordance with procedures approved by NYUAD's IRB.

### Task design

**Figure 1:**
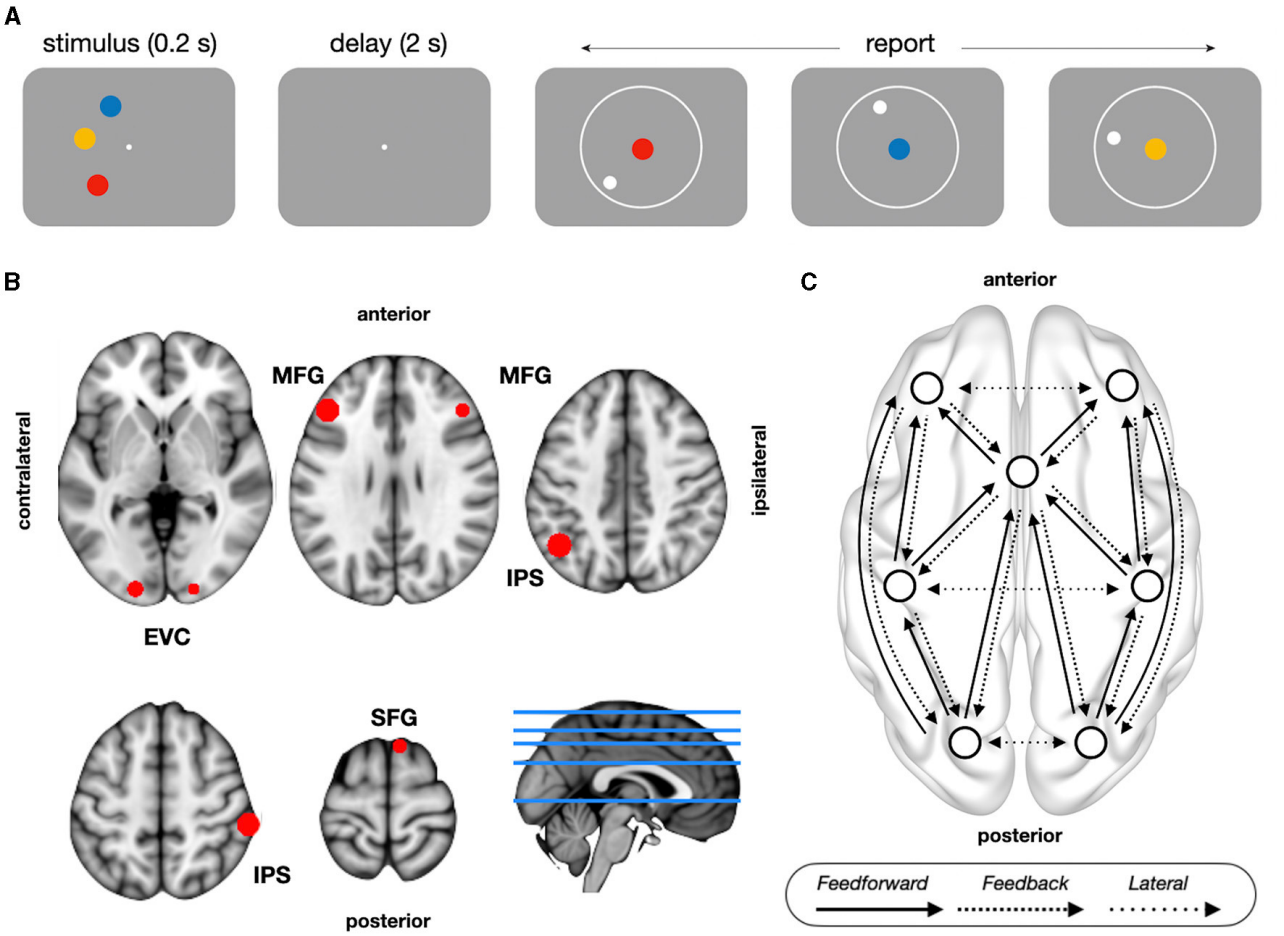
Task design and model architecture. **(A)** Delayed continuous report task. Subjects retained the colors and locations of three items over a memory delay and then responded indicating the location of each of the cued color items. **(B)** The location of sources used for the DCM analyses, as shown on an MNI template brain. **(C)** Schematic representation of model architecture with between-source excitatory connections. Feedforward connections (posterior-to-anterior) project from superficial pyramidal to spiny stellate cells; feedback connections (anterior-to-posterior) project from deep pyramidal to inhibitory interneurons and superficial pyramidal cells; lateral connections comprises both feedforward and feedback projections. EVC, early visual cortex; IPS, intraparietal sulcus; MFG, middle frontal gyrus; SFG, superior frontal gyrus.

Stimuli displays, timing, and responses were controlled and recorded using the Psychophysics Toolbox (Brainard, 1997) in MATLAB (The MathWorks, Inc., Natick MA, USA). We assessed WM behavior using a delayed continuous report task (Figure 1A). A fixation cross of 0.2 degrees of visual angle (DoV) was presented in the center of the screen during baseline, stimulus presentation, and memory delays. Each trial began with a 1 s fixation period, after which subjects saw three colored discs (each 0.55 DoV, presented at an eccentricity of 4.5 DoV) for 0.2 s. Subjects were asked to hold the colors and locations of all three discs over a blank 2 s memory delay. At the end of the memory delay, instead of asking subjects to report one of the items, we cued subjects to report the location of each of the three items sequentially (in a random order) by presenting a disc at fixation whose color indicated the color of the item subjects were to report. Subjects adjusted the position of a white circle of the same size and of the same eccentricity as the disc using an MEG-compatible response dial and locked in their report using a button box (Current Designs, Philadelphia PA, USA). Each location report was self-paced. Following all three reports, subjects were shown a 1 s feedback screen that indicated the presented and reported locations on that trial. The trial concluded with a self-paced intertrial interval (ITI)—that is, the next trial began when subjects pressed a button. Subjects completed 500 ± 75 (400–720) trials, divided into blocks of 80 trials (seven subjects) or 100 trials (19 subjects). Two subjects completed the experiment in two sessions 1 and 4 days apart; the remaining subjects completed the experiment in a single session.

The memory items were always presented in one hemifield in a given block of trials, and left and right hemifield blocks were presented in a random order. The color of the memory items on each trial were chosen from 180 color equally spaced segments drawn from a circle in CIELAB color space (radius 59°, centered at *L* = 54, *a* = 18, and *b* = −8) (CIE, 2004), with a minimum gap of 15 color segments between memory items on a given trial to avoid errors due to the color similarity of the memory items. Memory locations were randomly sampled from an isoeccentric (semi)circular space (depending on the hemifield block) with a minimum gap of 15° of polar angle. We additionally excluded locations that were 10° of polar angle away from the vertical meridian to avoid well-documented oblique effects (Furmanski and Engel, 2000).

Stimulus displays were projected onto a screen 85 cm away from the subject's head. Subjects were instructed to remain still during the MEG recordings and to avoid blinking or moving their eyes during the cue and delay screens. Subjects were free to move their eyes during the response screens and were encouraged to use the ITI as well as breaks between blocks to blink.

### Behavioral analysis

To label trials based on subjects' behavioral performance, we fit a probabilistic mixture model with three components (target, non-target, and guess responses) to the distribution of errors using the C016_fit function (currently called mixtureFit, bayslab.com) (Bays et al., 2009; Schneegans and Bays, 2016):


$$\begin{array}{l} p\left(\hat{\theta }\right)\;=\;\left(1-\;\beta -\;\gamma \right)\phi_{\sigma }\left(\hat{\theta }-\theta \right)+\;\gamma \frac{1}{2\pi }\\ \;+\;\beta \;\frac{1}{m}\;\sum_{i}^{m}\phi_{\sigma }(\hat{\theta }-\theta_{i}^{*}), \end{array}$$

where θ is the target location (in radians), $\hat{\theta }$ is the reported location, γ is the probability of random guess, β is the probability of swap, $\theta_{i}^{*}$ are the locations of non-targets with *i* = 1, …, *m* and *m* = 2, and ϕ_σ_ is the circular normal distribution (Von Mises) with zero mean and σ standard deviation. Maximum likelihood estimates of parameters σ, γ, and β were obtained at subject level. Then, trial-wise posterior probabilities that responses were drawn from each of the three mixture components were used to define trial types (see Figure 1C in Bays et al., 2009 and Figure 2 in Schneegans and Bays, 2016). High accuracy (correct) responses were trials with a target probability above 90% in all 3 reports, swaps errors were trials with a probability of non-target response above 70% in at least one report, and low accuracy trials comprised the remaining trials. The more lenient cut-off criterion for swap trials was motivated by the tendency of the model to underestimate swap frequency, while the conservative cut-off in correct trials in all three reports minimized the likelihood that correct trials contain lucky random guesses. Note that the label “low accuracy” does not mean that all three reports were inaccurate or guess responses; it simply indicates that subjects did not report all three items with a high degree of accuracy.

### MEG and MRI acquisition and preprocessing

**Figure 2:**
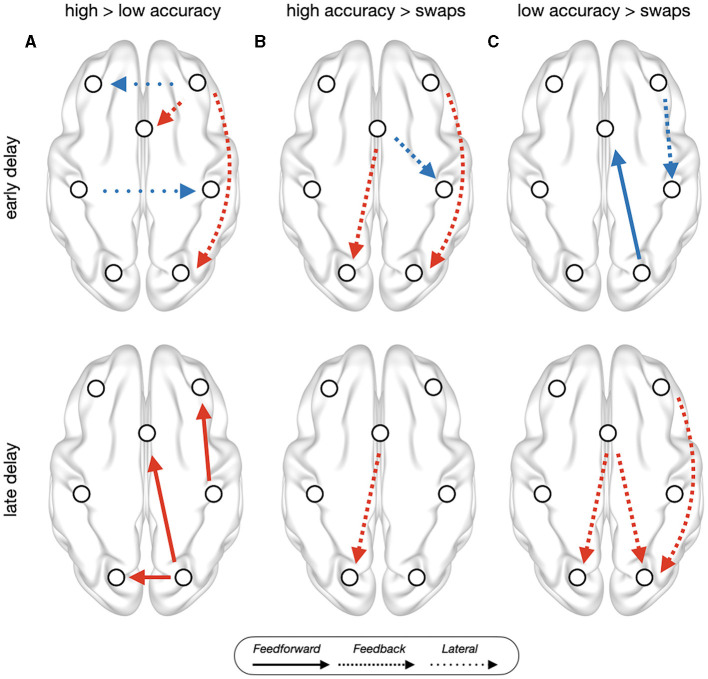
PEB results of trial type contrast between **(A)** high accuracy trials vs. low accuracy trials, **(B)** high accuracy vs. swap trials, and **(C)** low accuracy vs. swap trials, on the baseline corrected estimates of effective connectivity during early delay (top) and late delay (bottom). Red connections indicate trial-type1 > trial-type2, while blue indicate trial-type1 < trial-type2. All connections showed have posterior probability > 0.95 and out-of-sample predictive accuracy of *p*_uncorrected_ < 0.05. Left side corresponds to the contralateral hemisphere, anterior (frontal) brain areas are shown on top.

Before MEG acquisition, each subject's head shape was digitized using a Polhemus dual source handheld FastSCAN-II. MEG data was recorded continuously using a 208-channel axial gradiometer Yokogawa system (Kanazawa Institute of Technology, Kanazawa, Japan) with a sampling rate of 1,000 Hz and an online low-pass filter of 200 Hz. The continuous MEG data was first noise-reduced using eight magnetometer reference channels located away from the participant's head and using the Time Shifted Principle Component Analysis (TSPCA, block width of 5,000 and 30 shifts) as implemented in MEG160 software (Yokogawa Electric Corporation and Eagle Technology Corporation, Tokyo, Japan). To remove eye blink and heartbeat artifacts, an independent component analysis was performed with fast ICA method in MNE-Python (Ablin et al., 2018). All subsequent preprocessing steps were performed using FieldTrip (Oostenveld et al., 2011). Data was epoched from 1 s pre-stimulus onset to 2.7 s post-stimulus onset (including baseline, stimulus presentation, memory delay, and the first 0.5 s of response), demeaned, and low pass filtered at 140 Hz. In order to clean the data, we automatically rejected trials and channels with high variance using ft_artifact_zvalue.m function with the following settings: jumps artifacts threshold *z* = 20, muscle artifacts threshold *z* = 5 and blink artifacts threshold *z* = 10. Additionally, we manually inspected individual epochs to remove trials that were contaminated with jump, muscle, and eye blink artifacts. Rejected channels were reconstructed using the average of all neighbors. Preprocessing resulted in an average of 54 ± 37 (13–200) removed trials [accounting for the 11 ± 6% of total trials (2–29)], and 2 ± 1 (1–10) rejected channels (out of 208) per participant. The average number of trials available for analysis was 446 ± 69 (357–707): 106 ± 94 (19–439) (23 ± 17%) high accuracy trials, 213 ± 53 (80–328) (48 ± 11%) low accuracy trials, and 126 ± 46 (46–250) (29 ± 10%) swap trials. The experimental setup included trials with stimuli presented in either the left or right hemifield. To analyze all trials without canceling out lateralized neural signals, we took the following approach. For trials with left hemifield presentation, we mirrored (reflected) the MEG sensors across the midline, ensuring that MEG sensors on the left (right) side aligned with the hemisphere contralateral (ipsilateral) to the stimulus presentation (as depicted in Figure 2).

In a subset of eight subjects [age: 23.16 ± 3.54, range (20–31); two females, six males], we used a high-resolution anatomical MRI volume collected during a separate session to constrain the transformation from sensor to source space. The anatomical scan was acquired on a Siemens Magnetom Prisma 3T MRI scanner using a 6.5 min MPRAGE-3D T1-weighted, gradient-echo acquisition sequence (TR: 2,400 ms, TE = 2.22 ms, flip angle: 8 degrees, voxel size: 0.8 mm^3^, 208 slices, FOV: 256 mm).

### Selection of sources of interest

In order to study effective connectivity, we performed a time-frequency analysis in source space to identify the network of neural sources actively involved in WM, comprising frontal, parietal, and occipital regions. First, we localized oscillatory sources using the beamformer technique (Jaiswal et al., 2020; Westner et al., 2022). The head model template in MNI space (“standard_bem”) (Oostenveld et al., 2003) was transformed into a subject-specific head shape to compute the volume conduction model using a realistically shaped single shell approximation from FieldTrip's ft_prepare_headmodel.m function (Nolte, 2003). Next, we used ft_prepare_sourcemodel.m function to create a source model with a 1.25 cm isotropic 3D grid and with 1,499 sources within the head model. Finally, source and head models were used to create a forward model (ft_prepare_leadfield.m). For the subset of eight subjects with MRI data, the head model was computed using the structural T1 image, and the source model in standard space was spatially normalized into subject-specific structural image with a non-linear transformation (warp). As a result of this transformation, forward model positions were equivalent across subjects in normalized space. To compute the inverse solution, the cross-spectral density (CSD) matrix was calculated using discrete prolate spheroidal sequences (“dpss” taper in “mtmfft” method in ft_freqanalysis.m function) with all valid trials using baseline (−0.75 to −0.25 s) and delay period (0.2–2.2 s) epochs. The resulting spatial filter was used to localize sources in the frequency domain (computing power) using a beamformer dipole analysis with dynamic imaging of coherent sources (“dics” method in ft_sourceanalysis.m function) for frequencies from 2 to 100 Hz in steps of 2 Hz, with a smoothing of ± 2Hz, and a 5% regularization. For each trial type, source activity (power) during the delay period was computed in 31 time windows of 0.5 s, in steps of 0.05 s, and baseline-corrected (relative change method) with the trial-averaged baseline epoch of 0.5 s (−0.75 to −0.25 s, equally spaced from the beginning and end of the 1 s baseline). Finally, to normalize spatial maps across subjects, we transformed subject-specific maps of power from the head coordinate system to the MNI coordinate system using the inverse of the transformation matrix that was computed when generating the forward model.

Sources of interest for the DCM analysis were selected using a combined theory- and data-driven approach. Based on previous literature (Sreenivasan and D'Esposito, 2019), we predefined regions-of-interest (ROI) using the Brainnetome Atlas (Fan et al., 2016): superior frontal gyrus (SFG, areas 1–14), middle frontal gyrus (MFG, 15–26), intraparietal sulcus (IPS, 127–142), and early visual cortex (EVC, 203–206). Then, we used the time frequency matrices of power (31 time windows by 50 frequency bins) to select the sources of interest within each ROI. For each source, we performed a massive univariate one-way analysis of variance (ANOVA) with trial type as a factor with three levels (high accuracy, low accuracy, and swap trials), getting as output a time frequency matrix of *F*-statistics. Then, for each source, we summed the resulting uncorrected *F*-statistics over time and frequency, and selected the source with highest collapsed *F*-statistic within each ROI. All ROIs were considered separately for contralateral and ipsilateral hemispheres, except in SFG. There, given the proximity of the sources to the midline, we selected the source with the highest F-statistic bilaterally. Sources coordinates were (Figure 1B): SFG [*F*_collapsed_ = 3,576; MNI: (8, 18, 68)], MFG [contralateral: *F*_collapsed_ = 3,399, (−43, 30, 30); ipsilateral: *F*_collapsed_ = 3,760, (45, 30, 30)], IPS [contralateral: *F*_collapsed_ = 5,487, (−43, −58, 43); ipsilateral: *F*_collapsed_ = 4,129, (58, −33, 55)] and EVC [contralateral: *F*_collapsed_ = 3,108, (−18, −95, 5); ipsilateral: *F*_collapsed_ = 3,161, (20, −95, −8)]. Frontal and parietal nodes fall inside of a recent meta-analysis of fMRI studies about delay activity in WM (Li et al., 2022), while visual nodes fall in the occipital pole, from where previous studies decoded memory contents during delay period (Serences et al., 2009). Timecourses of selected sources of interest were reconstructed using a linear constrained minimum variance beamformer (“lcmv” method in FieldTrip) (Veen et al., 1997) with a fixed orientation projected along the first PCA axis.

### DCM model

We used a DCM with cross-spectral density as data feature (range 2–100 Hz in steps of 1 Hz), as implemented in SPM12 (Friston et al., 2012; Moran et al., 2013), with the canonical microcircuit model (CMM) based on the 4-population conductance-based neural mass model (Moran et al., 2011; Bastos et al., 2012). The model comprises two components: the neuronal model characterizes the dynamics of hidden neural states,


$$\dot{x}\begin{array}{l} =f(x,\;u,\;\theta )+\;\Gamma_{x}\; \end{array}$$

and the observation model describes how neural activity gives rise to observed data,


$$\begin{array}{l} y=h\left(C,\;x\right)+\;\epsilon \; \end{array}$$

where *x* is the hidden neural state, *u* is the inputs to the neuronal population, θ are the model parameters, Γ_*x*_ are the stochastic noise terms in neuronal model, *y* is the observed MEG data, *h* is the function mapping neural states to observed data, involving a lead matrix *C*, and ϵ are stochastic noise terms in the observation model.

In the conductance-based model used in the current study, neural dynamics are parametrized as the summed active and passive currents across the membrane (Pereira et al., 2021). Each neuronal population was modeled based on the Morris-Lecar model,


$$\begin{array}{l} C\dot{V}=\;\underset{k}{\sum }g_{k}\left(V_{K}-V\right)+u+\;\Gamma_{V}\; \end{array}$$

where *C* is the membrane capacitance, *V* is the membrane potential, *g*_*k*_ is the conductance of channel *k*, *V*_*K*_ is the reversal potential for channel *k*, *u* is the applied input current, Γ_*V*_ is the stochastic term modeling Gaussian noise. *k* comprises passive leak current, and active currents of excitatory (Na^+^) and inhibitory (Cl^−^) ion flow mediated by fast AMPA and GABAA receptors, depending on the type of neuronal population. Hence, membrane potential *V* and conductance of channels *g*_*k*_ comprise the hidden states *x* of the model in each neuronal population. Please refer to Pereira et al. (2021) for a full description of the mathematics of conductance based models, and see Moran et al. (2013) for a broad overview of neural mass models. Within each source (brain region), four neuronal populations are modeled: spiny stellate cells, superficial pyramidal cells, inhibitory interneurons, and deep pyramidal cells (Bastos et al., 2012). Neuronal populations within each source were connected as follows: (i) excitatory connections between excitatory spiny stellate and superficial pyramidal cells and from superficial pyramidal to deep pyramidal cells, (ii) recurrent connections between excitatory populations and the interlaminar population of inhibitory interneurons, and (iii) inhibitory self-connections of each population, modeling synaptic gain (Friston et al., 2019).

The connectivity between sources, the seven brain regions described above, was modeled through the matrices *A* = [*A*{1}, *A*{2}], the intrinsic connectivity matrices representing the strength of feedforward *A*{1} and feedback *A*{2} connections between different neural sources (see model architecture below). Types of connections were defined based on the specific populations they projected to. Feedforward connections originated from superficial pyramidal in the projecting source to spiny stellate in the target source, feedback connections originated from deep pyramidal in the projecting source to both inhibitory interneurons and superficial pyramidal cells in the target source, and lateral (interhemispheric) connection comprised both feedforward and feedback connections. Since our focus of interest was not the comparison between different model architectures of extrinsic connectivity (inference on model architecture), but rather the performance-dependent modulation of effective connectivity (inference on model parameters), we defined a model architecture with the canonical functional hierarchy. Building upon research on the functional organization of brain networks (Bastos et al., 2015; Michalareas et al., 2016; Nee and D'Esposito, 2016; Gratton C. et al., 2018; Gratton G. et al., 2018; Marek and Dosenbach, 2018) and previous DCM studies about WM (Dima et al., 2014; Heinzel et al., 2017), prefrontal cortex was placed at the top and visual cortex at the bottom of the hierarchy, designating posterior-to-anterior connections as feedforward, anterior-to-posterior connections as feedback, and interhemispheric connections between regions at the same level of the hierarchy as lateral connections (Figure 1C). Finally, the experimental effects of interests, the baseline corrected estimates of effective connectivity during delay period, were modeled with the matrix B, comprising all the connections defined in matrices A: 12 feedforward, 12 feedback, and six lateral connections (Figure 1C). Note that there is no circularity in using the same data to define ROIs and to study their interactions with DCM, since the purpose of DCM is to test hypothesis about the mechanisms underlying the experimental effects observed with conventional analysis (Stephan et al., 2010). Massive univariate statistical analyses (e.g., statistical parametric maps) to define ROIS are standard practice in several DCM studies (Moran et al., 2011; Dima et al., 2014; Auksztulewicz and Friston, 2015; Heinzel et al., 2017; Jung et al., 2018; Friston et al., 2019; Adams et al., 2021).

### Model inversion and comparison

For each subject, we inverted two DCMs for each trial type (high accuracy trials, low accuracy trials, and swap trials), encoding the baseline corrected estimates of effective connectivity (matrix B) during early and late delay. All time intervals had a length of 800 ms [baseline (−900 to −100 ms), early delay (400–1,200 ms), and late delay (1,200–2,000 ms)], and were separated from stimulus presentation (0 to 200 ms) or report (from 2,200 ms) by at least 100 ms. The separation of delay period in early and late time windows was motivated by previous studies in non-human primates showing that the dynamic properties of WM coding were stronger during the initial portions of the memory delay (Murray et al., 2017), and the modulation of memory load showed distinct spectral profiles during the early and late portions of the memory delay (Buschman et al., 2011) as well as differences in effective connectivity in frontoparietal networks (Pinotsis et al., 2019). Models were inverted to fit the cross-spectral density through the optimization of variational free energy under Laplace approximation (Friston et al., 2007) to optimize log scaling parameters around default priors (spm_fx_cmm.m). Please see Zeidman et al. (2023) for a comprehensive technical review of the Bayesian inference scheme employed to fit DCM models.

### Statistics over DCM parameters

We performed statistical inference on the parameters of the optimized DCM model using PEB (Friston et al., 2015; Zeidman et al., 2019). Compared to standard statistical inference, PEB has the advantage of taking into account the estimated uncertainty (variance) about model parameters, downweighing the contribution of less certain parameter estimates on group effects. The PEB approach is also advantageous in the context of model inversion and comparison, since the inversion of the same “full” DCM per subject/condition avoids the issue of fitting multiple models and performing Bayesian model comparison over models, which can lead to different DCMs falling into distinct local optima. PEB is a Bayesian hierarchical model over parameters with individual DCMs as a first level, and group effects on DCM parameters as a second level (see Zeidman et al., 2019 for details).


(1) $$\begin{array}{l} Y_{i}=\;\Gamma_{i}\left(\theta_{i}^{\left(1\right)}\right)+\;\varepsilon_{i}^{\left(1\right)} \end{array}$$

(2) $$\begin{array}{l} \theta^{\left(1\right)}=X\theta^{\left(2\right)}+\;\varepsilon_{i}^{\left(2\right)} \end{array}$$

The first level (Equation 1) represents the observed MEG data *Y* of the *i*-th subject modeled with the dynamic causal model Γ with parameters $\theta_{i}^{\left(1\right)}$ and noise term $\varepsilon_{i}^{\left(1\right)}$. The second level (Equation 2) shows that parameters of interest from first level θ^(1)^ are modeled at group level through a GLM with design matrix *X* and group-level parameters $\theta_{i}^{\left(2\right)}$, with zero-mean noise to account for between-subject variability as random effects $\varepsilon_{i}^{\left(2\right)}$. In our study, parameters of interest in PEB analysis (within-subjects design matrix) were the baseline corrected estimates of extrinsic (between-source) effective connectivity (matrix B). We performed pairwise comparisons between trial types with a group-level design matrix with an intercept (column of 1's) encoding commonalities, and a regressor of interest encoding the pairwise contrast between trial types (e.g., high accuracy vs. low accuracy). We report PEB parameters with strong evidence (posterior probability, *P*p > 0.95) (Kass and Raftery, 1995), and a large effect size, assessed through its predictive accuracy in a leave-one-out cross-validation (spm_dcm_loo.m). In this procedure, a PEB model was constructed with all subjects but one, predicting the excluded subject's covariates, and iterated through each participant serving as the left-out subject. The predictive accuracy was quantified as Pearson's correlation between PEB covariates and cross-validation predictions. We applied a statistical threshold of *p* = 0.05, uncorrected.

### Data and code availability

All scripts to invert DCMs and perform PEB analysis are available online: https://github.com/asantoangles/dcm_working_memory. Source timecourses used as input for DCM, in addition to inverted DCMs and PEB results, are available on Open Science Framework: https://osf.io/hbgu2/?view_only=8bc743e2068d47f0889f7b556beb2068.

## Results

### Behavioral results

Absolute mean error was 24.4° ± 5.17° (14.2°, 34.5°) for all trials, indicating that subjects were successfully able to complete the task. As expected by the definition of trial types, performance in high accuracy trials [11.2° ± 1.9° (7.4°, 16°)] was better than low accuracy trials [21.5° ± 4.9° [(14.3°, 33.8°); two-sample Wilcoxon test: *W* = 354, *z* = −6.12, *p* < 0.0001, effect size *r* = 0.85], and swap trials [51.6° ± 5.2° (43.4°, 68°); *W* = 351, *z* = −6.17, *p* < 0.0001, *r* = 0.85]. Performance in low accuracy trials was significantly better than swap trials (*W* = 351, *z* = −6.17, *p* < 0.0001, *r* = 0.85). Additionally, we observed no significant differences in absolute mean error between trials with left vs. right hemifield stimulus presentation [*W* = 734, *p* = 0.8; left: 25.2° ± 5.7° (13.2°, 36.5°), right: 23.7° ± 4.9° (13.9°, 32.5°)] and no difference in the proportion of left/right trials observed in distinct trial types (high accuracy trials: 0.49/0.51, low accuracy trials: 0.5/0.5, swap trials: 0.51/0.49; all pairwise comparisons between trials with a Chi-square test with Yates continuity yielded *p*-values > 0.8). The similar performance across hemifields helped justify our decision to flip sensors across the midline for MEG analysis (see Methods).

### DCM model fit

Correlation between empirical and predicted amplitude (modulus) was excellent (*r* = 0.9907 ± 0.004). Model predictions of within-ROI power spectral density (PSD) matched empirical data in all frequencies. Between-ROIs cross-spectral density (CSD) predictions were below the empirical values, although empirical CSD differences between trial types were generally reproduced by model predictions (Supplementary Figure S1B). Coefficient of determination also showed a good model fit [*R*^2^ = 0.97 ± 0.01 (0.46–0.99)]. PEB results summarized below describe the effects with strong evidence (P*p* > 0.95) an out-of-sample predictive accuracy with *p* < 0.05, uncorrected (see Supplementary material for a detailed description of PEB results).

### PEB results

Low accuracy trials, in comparison with high accuracy trials (Figure 2A), during early delay, showed reduced feedback signals in ipsilateral frontooccipital and medial to superior frontal connections, and increased interhemispheric connections between parietal and frontal areas. During late delay, low accuracy trials showed reduced feedforward signals in ipsilateral frontoparietal and frontooccipital connections, and reduced lateral connections between early visual areas. Swap trials, in comparison with high accuracy trials (Figure 2B), during early delay, showed reduced feedback connectivity in ipsilateral frontooccipital and increased superior frontal to ipsilateral parietal. Feedback connectivity was also reduced in superior frontal to contralateral early visual cortex during the whole delay period. Low accuracy trials, in comparison with swap trials (Figure 2C), during early delay, showed reduced feedback ipsilateral frontoparietal connectivity and reduced feedforward connectivity in ipsilateral early visual cortex to superior frontal. During late delay, low accuracy trials showed increased feedback connectivity in several frontooccipital connections: ipsilateral middle frontal to early visual cortex, and superior frontal to ipsilateral and contralateral visual areas.

## Discussion

In the current study, we report evidence that WM involves changes in effective connectivity across a network involving frontal, parietal, and visual areas; and demonstrated that these changes were performance-dependent. In comparison with high accuracy trials, low accuracy and swap error trials showed distinct patterns of effective connectivity. In early delay, both types of error trials showed reduced feedback connections in frontooccipital connections, but in late delay the pattern was qualitatively distinct: swap errors kept showing reduced frontooccipital connectivity, while low accuracy trials showed reduced feedforward connections emerging from early visual cortex and parietal to frontal areas. Taken together, our findings suggest that WM recruits a broad network of regions for optimal performance, and that errors in performance are associated with distinct systems-level reconfigurations of these networks, in agreement with the distributed view of WM (Lorenc and Sreenivasan, 2021; Mejías and Wang, 2022).

Cognitive functions emerge from a flexible reconfiguration of large-scale brain functional networks in response to task contingencies (Cohen and D'Esposito, 2016), with the integration of frontoparietal and visual networks (Spadone et al., 2015; Hearne et al., 2017). Here, we expand these previous studies by showing that large-scale network reconfigurations are performance dependent and actively involve early visual areas during WM maintenance. Our finding of reduced prefrontal feedback connectivity in error trials, both low accuracy and swaps, agrees with a DCM study of local field potentials in non-human primates during a change detection task (Pinotsis et al., 2019), where the authors reported an association between impaired behavioral performance and the break-down of prefrontal prediction signals in a network comprising prefrontal, frontal eyes field and lateral intraparietal area. We also observed reduced feedback connections between frontal nodes (middle to superior frontal in high vs. low accuracy), but most of the disrupted prefrontal feedback signals propagated to early visual cortex. Moreover, the specific connections involved depended on the type of error trials. While both low accuracy and swap error trials showed reduced feedback connections from middle frontal areas, feedback connections from superior frontal were also reduced in swap errors, but not in low accuracy errors. This pattern of performance-dependent disruption of feedback signals is consistent with the view that visual cortical engagement during WM at least partially reflects feedback signals from frontoparietal brain regions (Leavitt et al., 2017; Xu, 2020). At the same time, our observation of reduced feedforward signals in frontooccipital connections in low accuracy trials suggest that the role of visual cortex during the delay period is not solely that of a passive recipient of feedback signals, consistent with the sensory recruitment hypothesis of WM (Pasternak and Greenlee, 2005; D'Esposito, 2007; Scimeca et al., 2018).

Our findings revealed that network activity supporting WM maintenance conveys both feedforward and feedback signals involving not only frontoparietal networks (Dima et al., 2014; Heinzel et al., 2017; Jung et al., 2018), but also frontooccipital networks. Moreover, feedforward/feedback frontooccipital connections discriminated between types of errors. Swap error trials showed disrupted feedback connections to early visual areas, while low accuracy errors deployed a time-dependent pattern of effective connectivity changes of disrupted feedback (feedforward) signals in early (late) delay. This time-dependent disruption of effective connectivity in frontooccipital networks aligns with previous studies showing that the dynamic and spectral properties of WM coding differ between the early and late delay period (Buschman et al., 2011; Murray et al., 2017; Pinotsis et al., 2019). Moreover, it underscores the dynamic interplay between feedforward and feedback signals between early sensory areas and prefrontal regions, suggesting that WM is not a static storage system but a dynamically competitive interplay between sensory and cognitive control processes during different phases of WM maintenance. Interestingly, most of these connections involve (medial) superior frontal, a region previously associated in spatial WM (Courtney et al., 1998; Rottschy et al., 2012, 2013). Indeed, our results also suggest an alternative interpretation for previous findings of functional coupling of frontomedial theta (and alpha) with posterior brain regions during WM maintenance (Payne and Kounios, 2009; Hsieh and Ranganath, 2014; Johnson et al., 2017; Riddle et al., 2020; Parto Dezfouli et al., 2021; Ratcliffe et al., 2022). Although most studies have used correlational methods, this frontomedial activation during WM has been largely interpreted as control signals to posterior brain regions where information is stored (D'Esposito and Postle, 2015). Nevertheless, we found that the direction of effective connectivity involving medial prefrontal regions during WM depends on behavioral performance: medial PFC was the source of disrupted feedback signals in swap errors, but the target of disrupted feedforward signals in low accuracy errors.

Another distinction between types of errors was observed in the interhemispheric connectivity. Low accuracy trials showed increased lateral connectivity between frontal and parietal areas, which might be interpreted as system-level compensatory responses displayed in response to the absence of appropriate communication between occipital and frontal areas. Indeed, previous studies have shown that the experimental manipulation of interparietal communication affects WM performance (Tseng et al., 2018; Grover et al., 2022). Alternatively, it is plausible that an excessive level of interhemispheric communication has detrimental effects in WM performance (Koshy et al., 2020). On the contrary, swap errors showed no disruptions in lateral connections, consistent with the notion that swaps are genuine transposition errors arising from binding issues (Schneegans and Bays, 2017, 2019) and not merely educated guesses (Pratte, 2019; Huang, 2020). Otherwise, we would not see differences between types of error trials. Moreover, the absence of disrupted frontooccipital feedforward signals in swap trials aligns with the notion that transposition errors arise from incorrect responses being confidently held in WM (Schneegans and Bays, 2019). Memory contents can be decoded from early visual cortex in correct trials (Sreenivasan et al., 2014b; Curtis and Sprague, 2021; Lorenc and Sreenivasan, 2021), as well as in swap trials (Mallett et al., 2022). However, in swap trials the decoded information corresponds to the swapped location, not the cued location, suggesting the maintenance of incorrect memory items (Mallett et al., 2022). If we assume that feedforward signals propagating from early visual cortex during delay period distribute memory contents throughout the network, the non-disruption of feedforward signals in swaps might suggest that the incorrect information hold in early visual areas are propagated through the WM network in a similar way than in correct trials.

Empirical cross-spectral density revealed that coherence between sources accounted for most differences between trial types (Supplementary Figure S1A), despite the fact that sources were selected based on local power, supporting the behavioral relevance of large-scale communication through synchronization (Roux et al., 2012; Lisman and Jensen, 2013). Although trial type differences in coherence for some connections spanned the whole range of analyzed frequencies (2–100 Hz), the most striking differences were concentrated in low frequency bands, particularly around theta (6–8 Hz) and alpha (8–12 Hz), where we observed peaks in coherence (Supplementary Figure S1). These findings agrees with recent evidence that theta frequency synchronous tACS applied across frontoparietal network improves working memory performance, possibly by enhancing coherence between distant nodes of the network (Polanía et al., 2012; Violante et al., 2017; Biel et al., 2022; Grover et al., 2022).

Here, we acknowledge and address several potential limitations in our study. First, our definition of error trials was not entirely consistent with subjects' behavior, as low accuracy or swap trials were not exclusively determined by all three responses being misplaced or involving transposed reports, suggesting that not all memory contents was maintained poorly. Nevertheless, despite this limitation, we observed robust differences in effective connectivity between trial types, underscoring the robustness and validity of our findings. Second, the scope of our results is confined by our deliberate decision to limit the model architecture to frontoparietal and frontooccipital networks. Future investigations may consider incorporating nodes such as frontotemporal regions (Braun et al., 2015; Alenazi et al., 2022), salience and cinguloopercular regions (Cai et al., 2021), and exploring their interactions with the basal ganglia (McNab and Klingberg, 2008; Voytek and Knight, 2010). Third, our source reconstruction was performed without high-resolution structural images in a subset of subjects, thereby constraining our ability to make precise claims about the spatial location of sources. To address this spatial uncertainty, we employed predefined regions of interest and subsequently selected sources of interest within these regions that exhibited the most substantial main effect of trial type. To ensure the robustness of our source selection, we performed a supplementary analysis. Source reconstruction was carried out without structural images for all subjects, and euclidean distances between selected sources with and without a subset of participants with structural images were computed. The resulting distances of 11.2 ± 8.1 (0–18.4) (mm) were deemed negligible, given the spatial resolution of source-reconstructed MEG signals. Forth, an important consideration in our study is the observed imbalance in the number of trials between different trial types. However, the implementation of the parametric empirical Bayes (PEB) approach plays a pivotal role in mitigating the potential influence of this trial number asymmetry on our results. Unlike direct comparisons of effective connectivity estimates between trial types, our approach involved contrasting baseline-corrected estimates (matrix B). By focusing on these baseline-corrected values, our intention was to minimize the impact of trial number imbalances. This method emphasizes relative changes in connectivity patterns rather than absolute values, thereby contributing to the robustness of our findings. Fifth, the study utilized data from 26 subjects, comprising four women and 22 men. This gender mismatch presents a limitation in our investigation. Despite studies that have shown small (but significant) gender differences in WM performance, with the male/female advantage depending on domain and task (Voyer et al., 2017, 2021), there is no strong a priori reason to assume that similar behavioral performance should be supported by either the same or distinct underlying neural processes. However, gender differences in the functional dynamics supporting cognition are understudied. Preliminary evidence from task-based MEG studies in children and adolescents indicates potential sex-specific developmental effects during various cognitive tasks (Embury et al., 2019; Killanin et al., 2020, 2022; Taylor et al., 2020; Fung et al., 2021). MEG studies in adults have also shown gender differences neural responses during spatial navigation (Pu et al., 2020), face processing (Proverbio, 2021), and resting-state networks throughout the lifespan (Rempe et al., 2023; Stier et al., 2023). However, little is known about gender differences in effective connectivity, a gap we were unable to address with our current sample size. Future research should intentionally incorporate a more balanced representation of gender to elucidate potential gender-related effects on effective connectivity.

In conclusion, our study reveals performance-dependent reconfigurations in a large-scale brain network supporting WM. The network activity related to maintenance during the delay period conveys both feedforward and feedback signals, not confined solely to frontoparietal networks as depicted in the early work of Goldman-Rakic and colleagues. Instead, our findings extend this understanding to encompass frontooccipital networks, thereby emphasizing the crucial involvement of sensory areas in the dynamic processes of WM. Moreover, the disruption of these dynamics give rise to distinct types of behavioral errors. Low accuracy trials showed disrupted feedback (feedforward) signals during early (late) delay, while swap errors showed disrupted feedback signals during the whole delay period, aligning with the distributed view of WM.

## Data availability statement

Source timecourses used as input for DCM, in addition to inverted DCMs and PEB results, are available on Open Science Framework: https://osf.io/hbgu2/?view_only=8bc743e2068d47f0889f7b556beb2068.

## Ethics statement

The studies involving humans were approved by New York University Abu Dhabi Institutional Review Board. The studies were conducted in accordance with the local legislation and institutional requirements. The participants provided their written informed consent to participate in this study.

## Author contributions

AS-A: Conceptualization, Formal analysis, Investigation, Methodology, Writing – original draft. AT: Data curation, Investigation, Methodology, Project administration. VB: Data curation, Investigation. KS: Conceptualization, Funding acquisition, Project administration, Resources, Supervision, Writing – review & editing.
