# Supplementary material for: Effective connectivity of working memory performance: a DCM study of MEG data
Source: Front Hum Neurosci. 2024 Mar 4;18:1339728. doi: 10.3389/fnhum.2024.1339728 (PMC10944968; doi:10.3389/fnhum.2024.1339728)
Supplement: Supplementary file 1 [file Data_Sheet_1.docx]

**SUPPLEMENTARY MATERIAL**

**RESULTS**


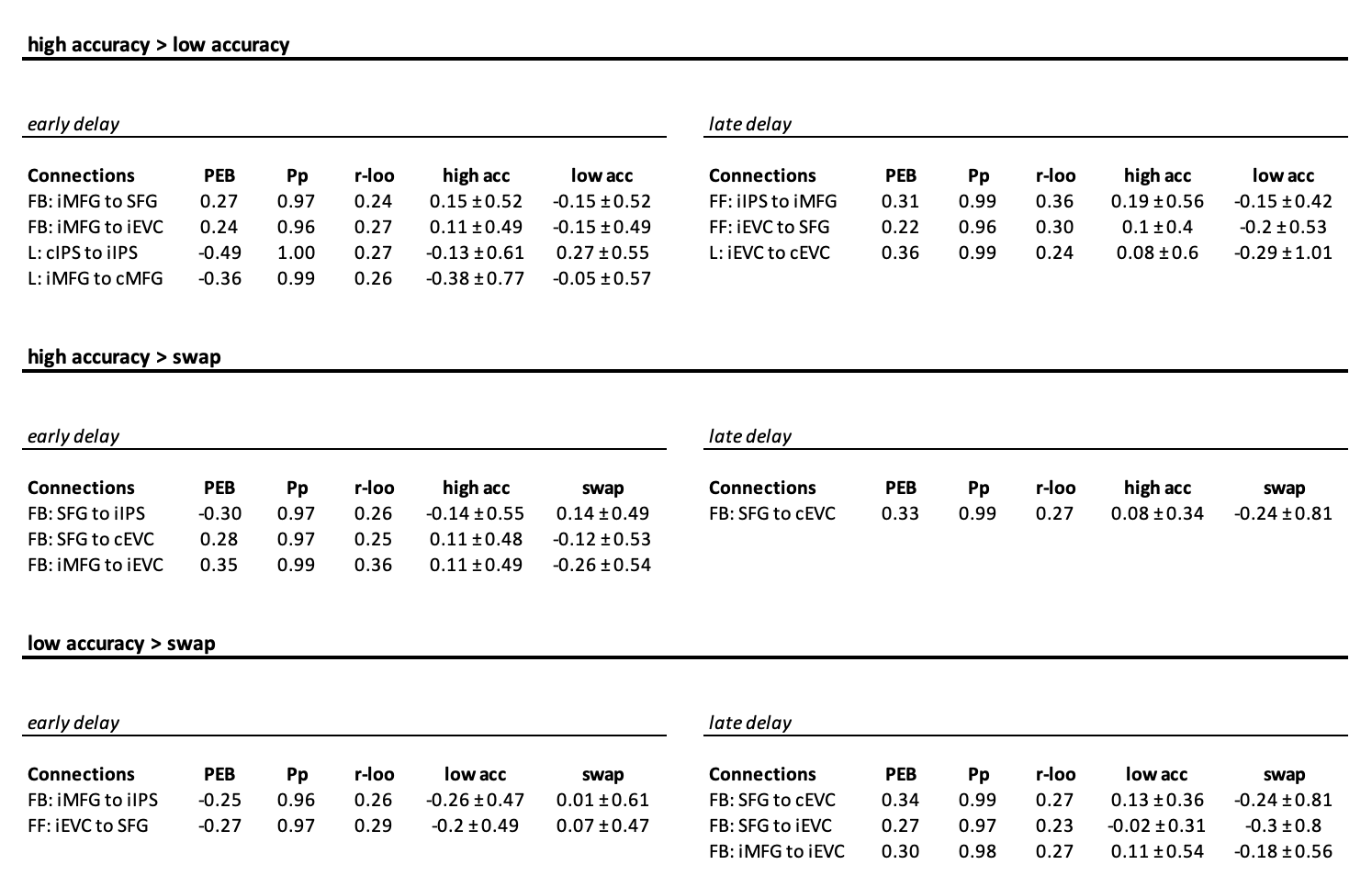


Table S1. Parametric Empirical Bayes (PEB) results of between trial type analysis in early and late delay, for the contrasts high accuracy vs low accuracy trials (top), high accuracy vs swap trials (middle) and low accuracy vs swap trials (bottom). Positive (negative) PEB values indicate trial type A>B (A<B) in the comparison. The fourth and fifth columns provide the mean and standard deviation across subjects of the extrinsic connectivity parameters (matrix B), encoding the baseline-corrected estimates of effective connectivity during delay period. Positive (negative) values indicate stronger (weaker) connectivity during delay period in comparison with baseline. cEVC, contralateral early visual cortex; cIPS, contralateral intraparietal sulcus; cMFG, contralateral middle frontal gyrus; FB, feedback; FF, feedforward; iEVC, ipsilateral early visual cortex; iIPS, ipsilateral intraparietal sulcus; iMFG, ipsilateral middle frontal gyrus; Pp, posterior probability; r-loo, correlation coefficient of the leave-one-out predictive accuracy; SFG, superior frontal gyrus.

**FIGURES**


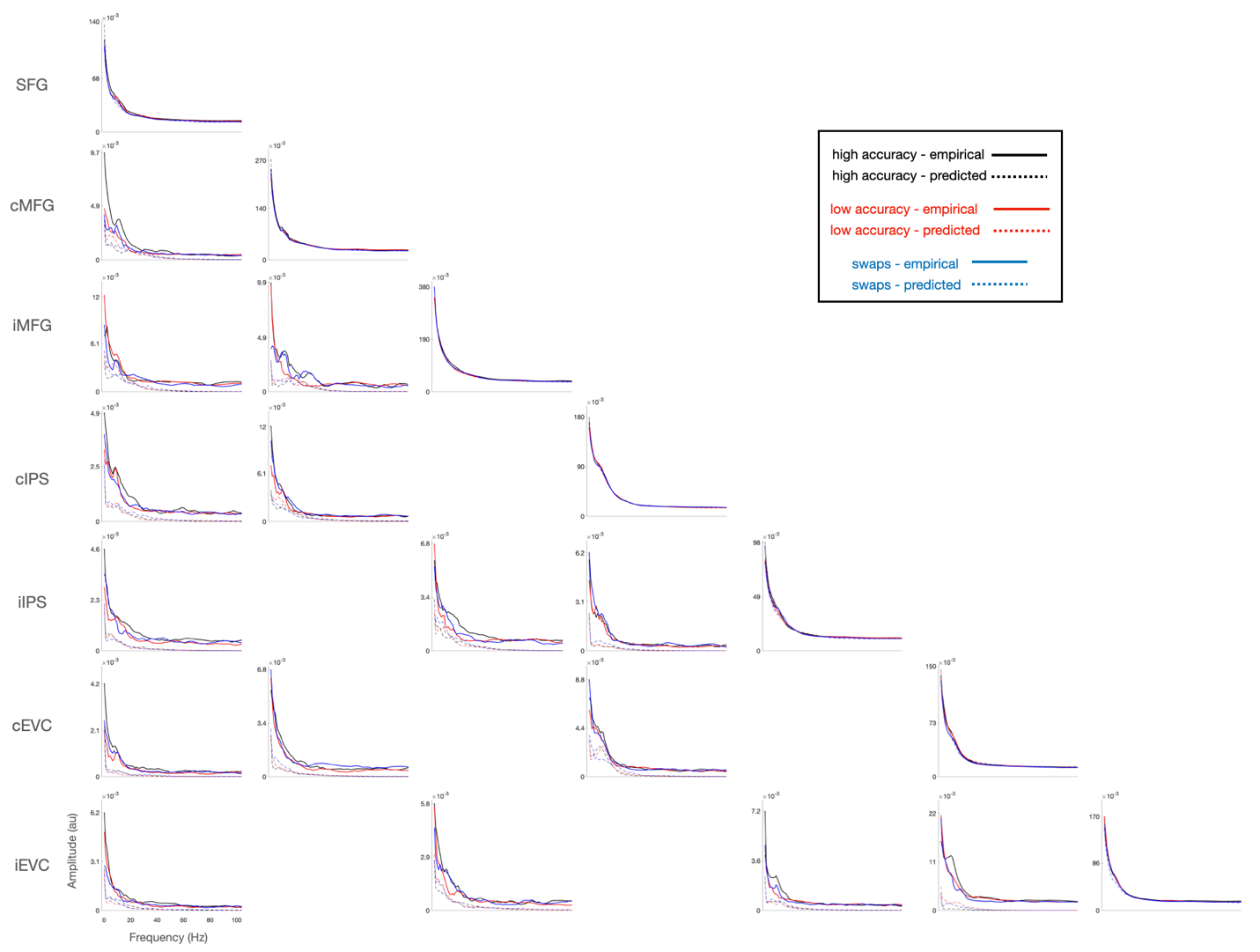


Figure S1. Empirical and predicted within-source power spectral density (on-diagonal plots) and between-sources cross-spectral density (off-diagonal), averaged across time windows, presented in matrix form. EVC, early visual cortex; IPS, intraparietal sulcus; MFG, middle frontal gyrus; SFG, superior frontal gyrus.
